# State of the “Art” in Freezing of Gait: Drawing on Perspectives of Individuals With Parkinson's Disease

**DOI:** 10.1111/ejn.70714

**Published:** 2026-10-12

**Authors:** Monika Pötter‐Nerger, Blanca T. M. Spee, Molly Zeitzschel, Neele Siems, Alessandro Gulberti, Erika Howe, Kaylena A. Ehgoetz Martens, Moran Gilat, Alice Nieuwboer, Bastiaan R. Bloem

**Affiliations:** ^1^ Department of Neurology University Medical Center Hamburg‐Eppendorf Hamburg Germany; ^2^ Department of Neurology Radboud University Medical Center, Donders Institute for Brain, Cognition and Behavior, Center of Expertise for Parkinson and Movement Disorders Nijmegen the Netherlands; ^3^ Department of Cognition, Emotion, and Methods in Psychology, Faculty of Psychology University of Vienna Vienna Austria; ^4^ Neurocognition and Mobility Laboratory, Department of Kinesiology and Health Sciences University of Waterloo Waterloo Ontario Canada; ^5^ Department of Rehabilitation Sciences Research Group for Neurorehabilitation (eNRGy), KU Leuven Leuven Belgium

**Keywords:** drawing of freezing of gait, emotions, lived experience, painting, Parkinson's disease, perspective by individuals with Parkinson's disease

## Abstract

Freezing of gait (FoG) in Parkinson's disease (PD) is increasingly recognized as a multidimensional phenomenon encompassing motor, cognitive, and emotional domains. The impact of FoG is enormous, but can be difficult to articulate. Traditional clinical assessments typically fail to capture its subjective and affective impact. This qualitative study explored drawings from individuals with PD to better understand the lived experience of FoG. Specifically, we collected artworks created by individuals who experience FoG. We studied the personal perceptions and emotions associated with FoG drawings in two ways: by asking participants to narratively explain their artwork and a qualitative curation by an international panel of experts of neurology, neuroscience, neurorehabilitation, neuroaesthetics, art, and empirical aesthetics. Five overarching themes emerged: (1) “Captured feet,” depicting sensations of being glued, tethered, or rooted to the ground; (2) “Chaos,” reflecting disrupted motor control during environmental or cognitive overload; (3) “Emotional explosion,” expressing fear, anger, helplessness, and loss of control; (4) “Freezing and falls,” highlighting instability and existential threat; and (5) “Released from freeze,” portraying therapeutic relief, restored movement, and joy. These visual narratives reveal that FoG is more than just a motor block. Rather, it is an embodied experience of immobilization, emotional escalation, and vulnerability, contrasted by profound relief when mobility returns. Artistic expression offers unique insights beyond structured assessments, fostering empathetic clinician–patient communication, and supporting a more holistic, person‐centered approach to PD care.

## Graphical abstract

Patient‐created drawings reveal that freezing of gait in Parkinson's disease is more than a motor block: an embodied experience of captured feet, chaos, emotional explosion, and fear of falling. The artworks also depict therapeutic release and joy when movement returns, highlighting how artistic expression can enrich clinician–patient communication and support more person‐centered care.

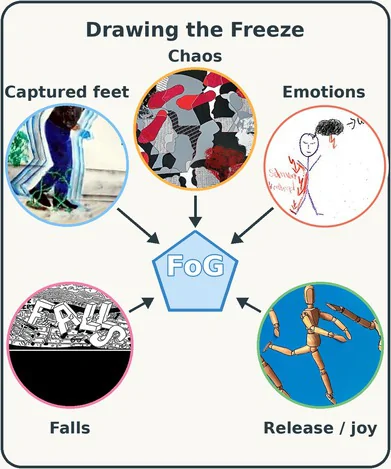

**Abbreviations**
FoG: freezing of gait
PD: Parkinson's disease

## 1 Introduction

Nir Giladi was among the first to systematically characterize freezing of gait (FoG) in Parkinson's disease (PD). He published seminal studies that described its clinical features, prevalence, and impact on daily lives in individuals with PD. His work laid the foundation for decades of research into both motor and nonmotor aspects of this complex phenomenon (Giladi et al. 1992; Giladi 2001; Giladi, McDermott, et al. 2001; Giladi, Treves, et al. 2001; Giladi and Nieuwboer 2008). He was involved in the new updated definition of FoG that reads as “paroxysmal episodes wherein there is an inability to step effectively, despite attempting to do so” (Gilat et al. 2026). Early on, Nir Giladi highlighted the subjective aspects of FoG, noting individual descriptions from people with PD as sensations of “blocks” or “feet seemingly glued to the ground.” He linked FoG to impaired quality of life, fear of falling, and loss of independence, beyond what is captured by purely motor assessments (Giladi and Hausdorff 2006; Moore et al. 2007).

Since then, the close interplay between FoG and both emotional experiences (Witt et al. 2019) and cognitive processes (Monaghan et al. 2023) has remained the focus of research, even leading to a potential classification of FoG subtypes (Ehgoetz Martens et al. 2018). However, traditional clinical assessments often fail to capture the broad and subjective impact of FoG that is uniquely felt by those who experience this phenomenon (Mancini et al. 2019). This is particularly true for the complex feelings of frustration, fear, or loss of control that typically accompany FoG episodes.

Visual art, including patient‐created drawings, offers a unique window into these experiences (Cheung et al. 2016; Broadbent et al. 2019). Using art to express emotions on disease symptoms has already been used in other disease conditions such as asthma (Cheung et al. 2020), lupus erythematodes (Nowicka‐Sauer 2007), heart failure (Reynolds et al. 2007; Princip et al. 2015) or headache (Broadbent et al. 2009). Case studies have reported that drawings created by individuals with PD provide general insight into the neurobiological linkages between the mind, brain, body, and the human penchant for art‐making itself (Pelowski et al. 2022). However, there is limited literature on the use of art to explore patients' experiences of specific symptoms and their associated emotional impact. One recent study reported art collections of emotions depicted by individuals with PD wo had received deep brain stimulation (Chacón Gámez et al. 2021), but to date, there is no published work on the emotions of individuals with PD captured through drawings specifically related to FoG.

The aim of this drawing collection from individuals with lived experience with PD and FoG is to examine these visual representations of FOG, in order to identify recurring themes, metaphors, and emotional signatures that capture diverse lived experiences with FoG.

## 2 Methods

This study was designed as a qualitative, exploratory investigation. The study was conducted in accordance with the ethical standards of the 1964 Declaration of Helsinki and its later amendments. Ethical approval was obtained and approved by the Ärztekammer Hamburg, Germany (2025‐101589‐BO‐ff).

### 2.1 Participants

Participants were eligible if they had a diagnosis of PD, were aged ≥ 18 years, were able to provide written, informed consent and were able to offer one or more drawings related specifically to FoG. No personal data were collected or processed besides the art pieces and their interpretations by the individuals with PD.

### 2.2 Procedures

#### 2.2.1 Drawing Acquisition

The drawings included in this study were obtained through three distinct modes of acquisition, which we now designate as categories A, B, and C.

 A: Drawings were collected during inpatient stays at the University Medical Center Hamburg‐Eppendorf, Hamburg, Germany. To facilitate expression, participants at the German study site received a worksheet containing open‐ended guiding questions, such as how FoG feels in their body, which color or weather condition might represent it, how a “before‐during‐after” sequence of an episode could be illustrated, what their legs might “say” during a freezing episode, and how treatments such as levodopa or deep brain stimulation influenced their symptoms. The instructions were intentionally open to encourage authentic and individualized expression. The drawings were subsequently discussed during clinical grand rounds with the corresponding author with regard to their possible interpretations.
 B: Drawings were solicited via a call distributed by email through the patients supporting groups “German Parkinson's disease association” and “PingPongParkinson.” Individuals with PD submitted their drawings directly to the corresponding author via email, who subsequently discussed the meaning and interpretation of each drawing with the respective participant via email.
 C: Drawings were collected in the Netherlands.

For each artwork shown in Figures 1, 2, 3, 4, 5 and Figures S1–S3, the category of provenance (A, B, and C) is supplied in the figure legends.

In all three categories, participants were invited to create an artwork depicting their personal experience with FoG. Participants were reassured that there was no right or wrong way to depict their experiences and that the emphasis lay on the creative process rather than artistic quality. Metaphorical representations were encouraged. Individuals with PD were asked to explain the meaning of their drawings to ensure that their intended message was clearly and accurately conveyed. Some of the individuals offered more than one drawing on the FoG topic, aiming to shed light on different aspects of FoG. One individual brought a large piece of cardboard, which had been painted with various sketches on different FoG themes, which were then divided into separate chapters according to the intended meaning that was discussed in longer, personal conversations.

Drawings were collected between August 2025 and December 2025. Thus, the dataset combined newly elicited artworks from the German study site with pre‐existing artworks collected in the Netherlands.

#### 2.2.2 Drawing Selection and Categorization

The international panel from Nijmegen, Leuven, Waterloo, and Hamburg consisted of experts with different professional backgrounds (neurology, neuroscience, neurorehabilitation, neuroaesthetics, art, and empirical aesthetics). The analytic approach was an exploratory, panel‐based curatorial interpretive synthesis rather than a formal, grounded‐theory, quantitative protocol. The panel considered each artwork together with its title and the participant's accompanying explanation, selected images on the basis of the core message they were intended to convey, and grouped recurrent experiential content into five overarching themes. The unit of interpretation was the participant‐artwork account rather than an assumption that each artwork represented an independent observation. Because several participants contributed more than one artwork, repeated contributions were treated as complementary depictions of that participant's experience and not as additional independent support for a theme. The study did not use a formal coding framework, quantitative theme frequencies, independent coder reliability testing, a prespecified disagreement‐resolution procedure, or a formal audit trail. Participants' own explanations were used throughout to constrain interpretation, and the first manuscript version was returned to participating individuals to ascertain consent. Thus, the five themes need to be understood as an interpretive organization of this collection rather than validated or prevalence‐weighted categories.

Prompts to and interpretations provided by individuals with PD were communicated in their respective native languages, either Dutch or German. All non‐English material was subsequently translated into English. To ensure that the translations accurately reflected the participants' intended meanings, the relevant manuscript content was shared with the respective participants for review and confirmation.

## 3 Results

Thirty artworks from 18 individuals with PD were selected for this report (see main text and Supporting Information) and summarized into five overarching themes by recurrent experiential content across the collection.

### 3.1 “Captured Feet”

One prominent and repeatedly expressed theme was the experience of the “Feet being captured” during episodes of FoG (Figure 1). In his drawing entitled “Tethered to earth,” (Figure 1, left column), M.H. depicted his legs entangled in vines while he is approaching an archway, his movement trajectory blocked illustrated by blue bodily forms. He described the image as follows: “The picture shows how my legs are being ‘held in place’ by vines—that's how it feels to me when things get tighter and I become blocked. The gate also has its meaning. The narrower the passage, the harder it is for me to get out of the freezing.” His description highlights both the sensory‐motor phenomenon of being physically restrained and the situational worsening of freezing in narrow or constricted environments—circumstances well known to provoke FoG. Similarly, C.B. titled his artwork “Feet nailed to floor” (Figure 1, middle column) and added the brief yet powerful statement: “Why me, what's wrong.” The image and accompanying words convey not only the bodily sensation of immobility but also the emotional burden and existential questioning associated with these episodes. In his picture “Captivated” (Figure 1, right column), R.H. illustrated the experience of being bound within chains like in the myth of Prometheus. He explained: “For me, freezing is the feeling of being rooted, tied up, blocked, and helplessly exposed to the holding forces, from which one can only free oneself with great effort. Alongside this is the fear that the initial hopping movements when starting to walk could lead to a loss of balance and, consequently, a fall.” His account emphasizes the multidimensional character of FoG, motor blocks during gait initiation, insecurity during ambulation, compensatory movements, and a persistent fear of falling. In summary, individuals with PD describe an overpowering downward or restraining force that prevents forward progression despite the intention to walk. This theme was further illustrated in Figure S1. A.S. portrayed his feet as “Feet bound by rubber bands” or M.N. as “Gravity of Gait,” who portrayed his feet simultaneously attempting to move forward while being pulled downward into the depth of earth with equal force. M.N. described his drawing: “Freezing feels like as if weights were pulling at my feet forward while sinking them at the same time downward into the ground.” Across these depictions, a common experiential core emerges, namely the sensation of the feet being glued, nailed, rooted, or tethered to the ground resulting in walking blocks.

**FIGURE 1:**
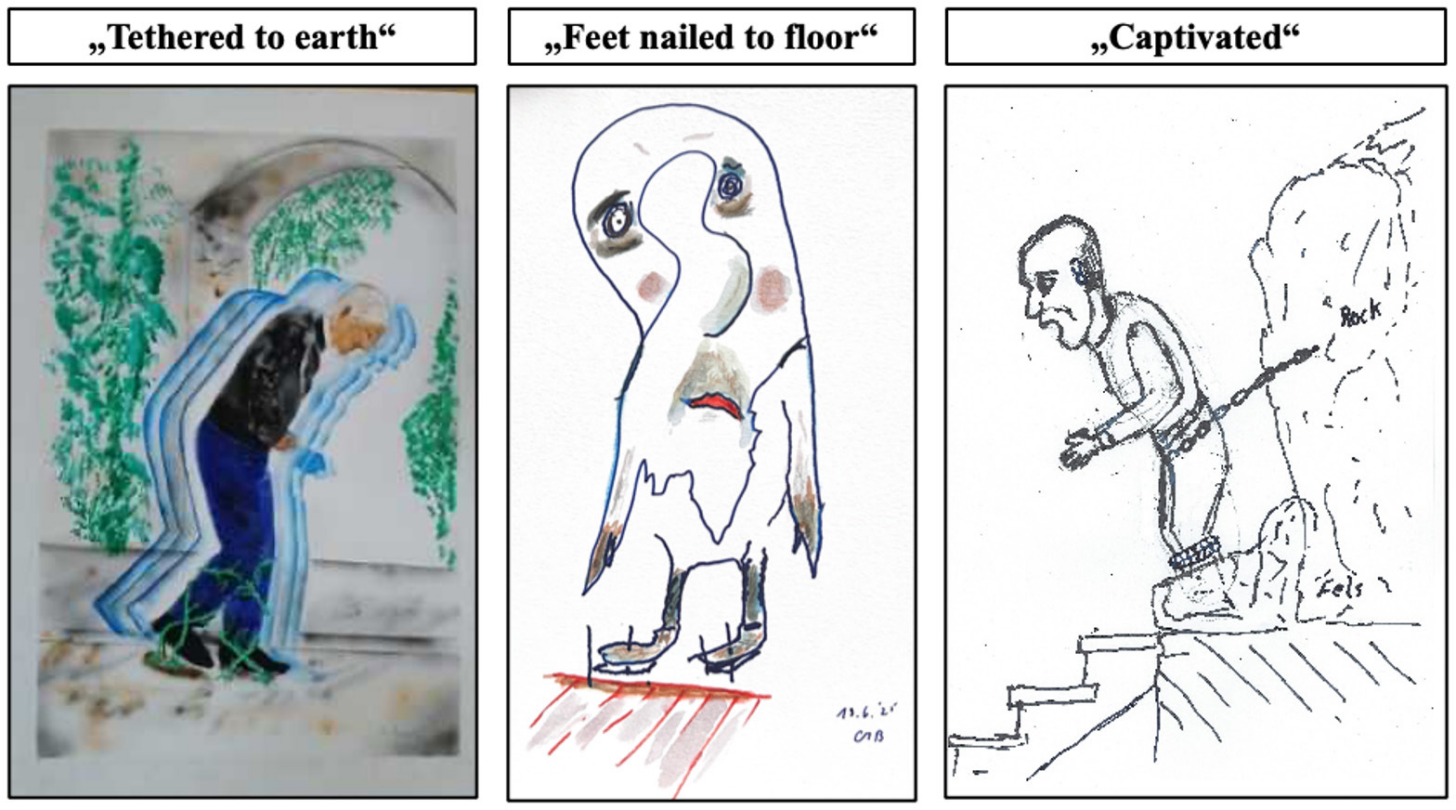
“Captured feet.” Individuals with Parkinson's disease described freezing of gait episodes as being “tethered to earth” (category B), having “feet nailed to the floor” (category B), or feeling “captivated” (category B), illustrating the profound perceptual and feet‐centered bodily dimensions of gait arrest. *Explanation of categories of Drawing acquisition: category A: Drawings were collected during inpatient stays at the University Medical Center Hamburg‐Eppendorf, Hamburg, Germany, in response to prompts designed to stimulate creativity, strengthen self‐confidence. Category B: Drawings were solicited via email through the patients supporting groups “German Parkinsons disease association” and “PingPongParkinson.” Category C: Drawings were collected in the Netherlands.

### 3.2 “Chaos”

A second overarching topic that emerged from the artwork was the theme of *chaos* (Figure 2) as a recurrent and defining experience during freezing episodes. In contrast to the sensation of being physically tethered to the ground, this theme reflects a subjective loss of order—both in the surrounding environment and within motor control itself. In his drawing entitled “The Way (Out?),” K.S. describes the transformation of walking from stability into disorganization: “At first, walking feels like a straight path that is well paved. But then the path becomes bumpy, uneven, irregular, chaotic, the paving stones are no longer symmetrical but asymmetrical and jumbled together. One wants to find a way out, symbolized by doors along the path, and to bring the freezing to an end” (Figure 2, left column). The metaphor of the path vividly conveys the disruption of rhythmicity and predictability that characterizes freezing. What begins as a structured and manageable trajectory becomes fragmented and chaotic. The doors along the path symbolize the search for resolution—an attempt to restore coherence and regain forward movement. S.W.‐T., in her artwork “Frozen in the midst of many,” emphasizes environmental overload as a precipitating factor (Figure 2, right column): “Chaos stops the steps. The crowd, the heads, the people, the voices is overwhelming the mind and then the feet get stuck. The heads mean that freezing occurs in a busy environment with many people, lots of voices, and a lot of chaos.” Her depiction underscores the interaction between cognitive overload and motor arrest. The multitude of faces and voices represents sensory and attentional demands that exceed processing capacity, culminating in a breakdown of gait initiation and continuation. Freezing, in this context, emerges as the motor expression of overwhelmed cognitive control. In his picture *“Insubordination*” (Figure S1), C.B. highlights an internal dimension of chaos: “The legs feel like a refusal to obey my orders.” Taken together, these accounts portray FoG not only as motor blockade but as a state of disintegration—of orderly pathways becoming irregular and noisy, of environments becoming overwhelming, and of motor commands losing authority. Chaos, as described by the individuals, is both external and internal, it arises from environmental complexity and from a disruption in the integration of cognitive, sensory, and motor processes required for fluent walking.

**FIGURE 2:**
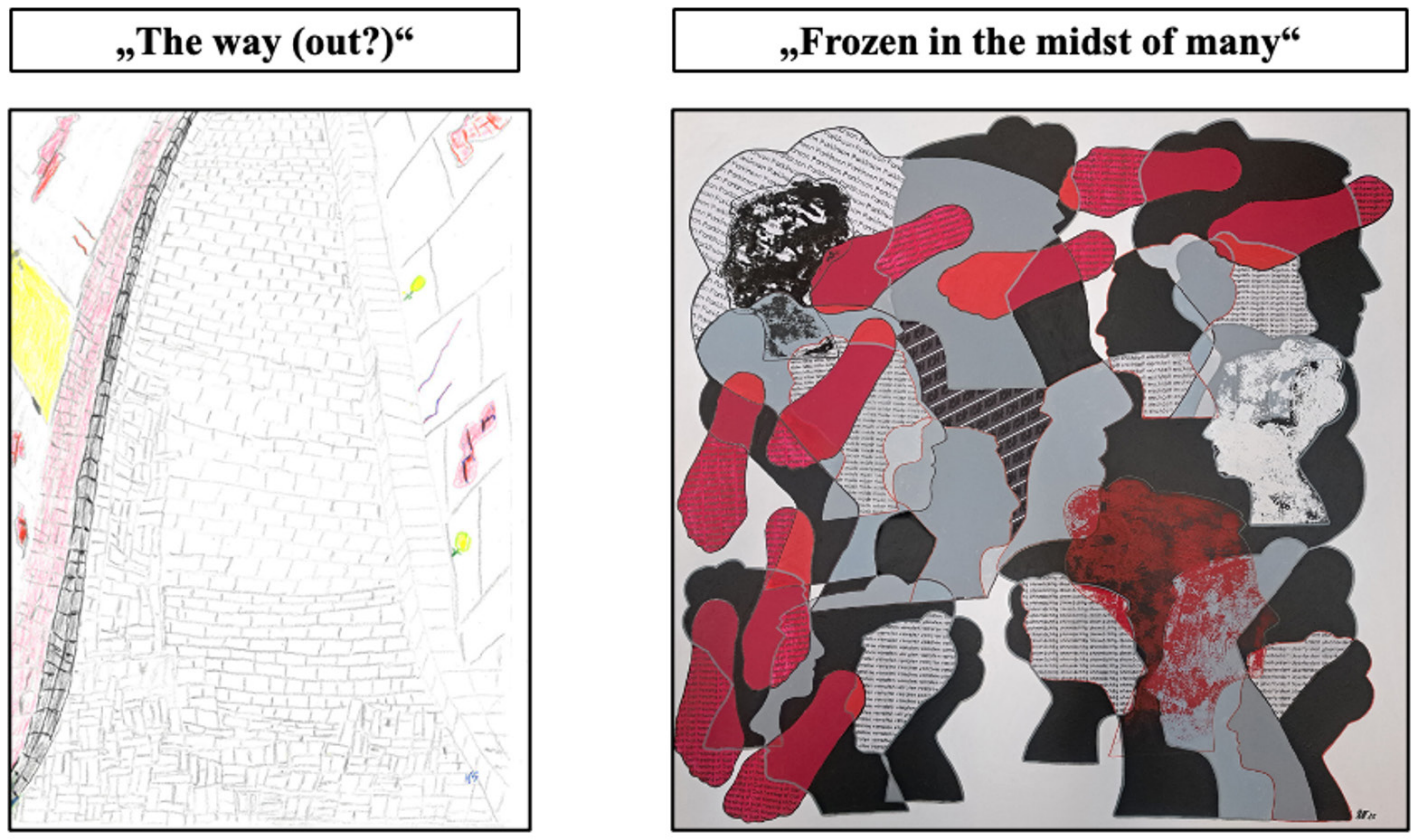
“Chaos.” Artistic depictions of the disorientation and perceptual overload associated with freezing of gait in Parkinsons disease. Patients portrayed the search for “The way (out)?” (category A) as a fragmented spatial experience and described being “Frozen in the midst of many” (category B), reflecting impaired navigation, sensory overcrowding, and the feeling of immobilization within complex social and environmental contexts. *Explanation of categories of Drawing acquisition: category A: Drawings were collected during inpatient stays at the University Medical Center Hamburg‐Eppendorf, Hamburg, Germany, in response to prompts designed to stimulate creativity, strengthen self‐confidence. Category B: Drawings were solicited via email through the patients supporting groups “German Parkinsons disease association” and “PingPongParkinson.” Category C: Drawings were collected in the Netherlands.

### 3.3 “Explosion of Emotions”

A third overarching theme that emerged from the artwork of individuals with PD can be summarized as an *“*Explosion of emotions” (Figure 3). In contrast to the previously described themes of physical fixation and chaos, these images foreground the intense affective dimension of FoG. Individuals describe freezing not merely as motor arrest, but as a state of escalating inner tension, emotional overwhelm, and abrupt loss of control. E.K. illustrates in her picture, “Freezing ride of emotions,” how her experiences of FoG feel like riding a roller coaster: “Freezing feels like riding a roller coaster. In the first moment you think you can make it, then suddenly you feel like all you can do is scream.” Her description conveys the unpredictability and rapid emotional escalation during freezing episodes with the sudden shift from confidence to helplessness, depicting the abrupt onset characteristic of FoG. Similarly, one individual with PD writes about her drawing “Frozen in fury”: “Freezing feels like my body betrays me. I feel strong anger, pain and cramps.” This illustrates how motor arrest is accompanied by affective distress and muscular cramping, reinforcing the sense of being overpowered by one's own body. K. B. in his artwork “Ballooned to freeze,” describes a mounting internal pressure: “Living with freezing of gait feels like a balloon being inflated—more and more—until finally, the balloon bursts. In that instant, movement stops, tension explodes, and all control feels lost.” This metaphor of inflation and rupture vividly depicts the buildup of psychological and physical strain culminating in abrupt immobility. R. L. in “Everything gray – the moment freezes,” emphasizes the internal and cognitive dimension: “The Freezing happens in the head. All of this represents the thoughts and feelings of the vaguely discernible head that surrounds the figure. A rigid shadow, moving sluggishly, like in a space suit, from the cold, rigid presence reached the back toward the warm light.” His imagery suggests emotional withdrawal, rigidity, and detachment. The contrast between cold rigidity and distant warm light may symbolize the struggle between immobility and the desire for movement, between emotional numbness and vitality. Further drawings on emotional distress associated with FoG are shown in Figure S2. N. E. in “The red barrier” portrays freezing as an invisible obstruction: “Freezing is like an invisible, red barrier–nothing works anymore. I then wait for movement as if I were in a ‘waiting mode,’ like a phone that is waiting for a signal or WIFI, as if there was no connection to the body yet.” Her comparison to a device awaiting connection reflects a perceived disruption between intention and bodily execution. The emotional tone is one of suspension and frustrated anticipation—being present yet unable to act. Finally, J.A. uses humor and visual wordplay in his cartoons “Gait” and “Freezing.” Regarding “Gait,” he explains: “This image uses a visual homophone to emphasize that walking isn't straightforward, and there can be barriers.” About “Freezing,” he notes: “This image uses the concept that when someone freezes, it can feel like a long time to unfreeze.” Through visual metaphor and temporal exaggeration, these cartoons capture both the obstruction of movement and the subjective distortion of time experienced during freezing episodes. The temporal distortion and subjective exaggerated slowness of movements was also captured by the drawing “Left back”: “Everyone moves ahead quickly as if in a car, while I am left behind—slow like a lagging pedestrian. I run after them, yet I am still left back.” E.K. and K.B. compare FoG episodes to stormy weather conditions, describing them as akin to blizzards or thunderstorms (Figure S1). Across these artistic representations, freezing emerges as more than a motor symptom. It is portrayed as an emotionally charged event marked by escalating tension, anger, fear, helplessness, and loss of control. The “explosion” described by individuals with PD reflects the convergence of motor arrest with intense affective and cognitive reactions. Together, these accounts underscore that FoG is not solely a disturbance of locomotion, but a profound embodied experience in which movement, emotion, and self‐perception are deeply intertwined.

**FIGURE 3:**
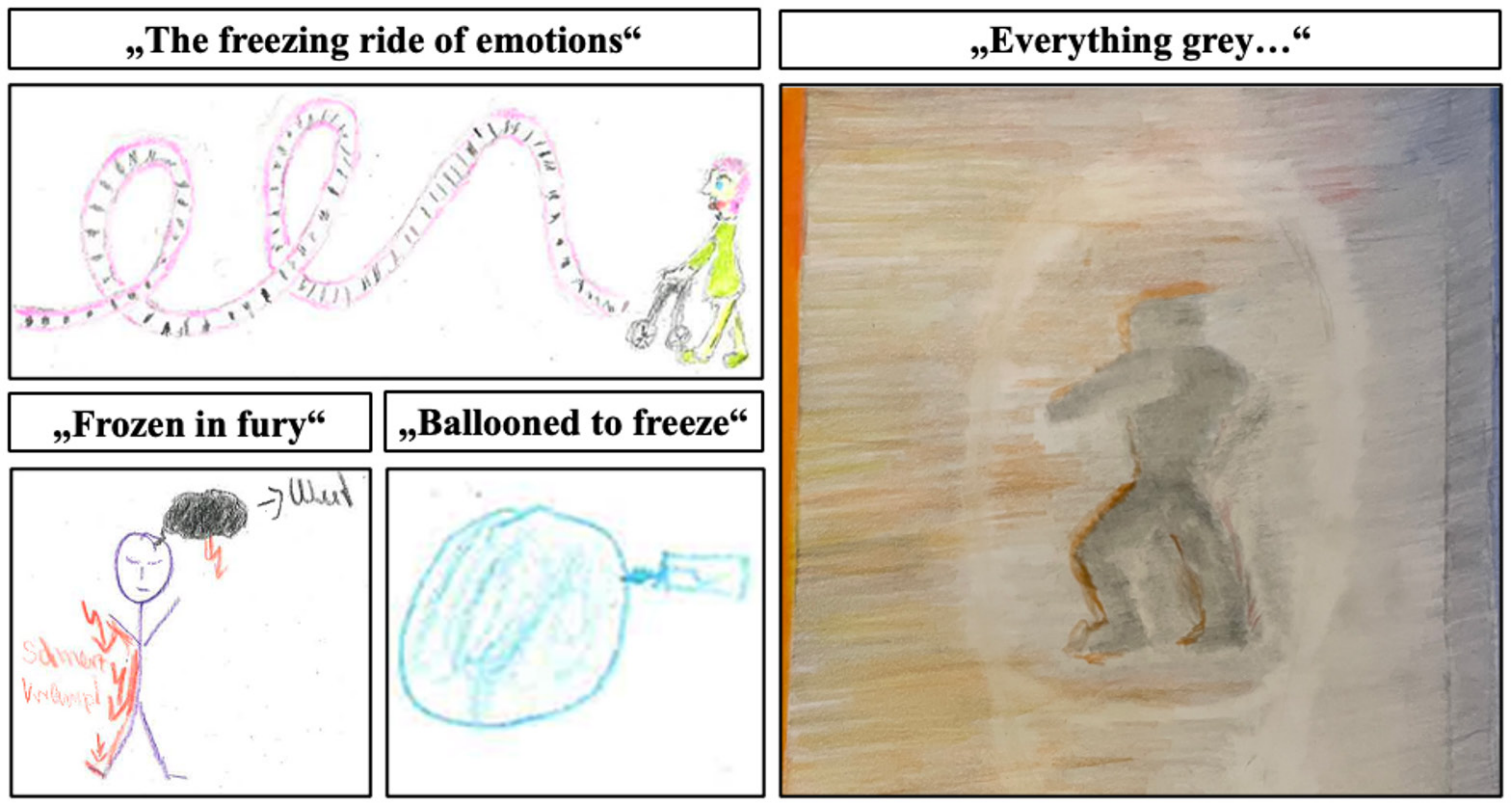
“Explosion of emotions.” Artistic representations of the emotional burden associated with freezing of gait in Parkinsons disease. Patients expressed freezing as an overwhelming cascade of emotions as uncertainty in “The freezing ride of emotions” (category A), anger in “Frozen in fury” (category A), progressive inner tension in “Ballooned to freeze” (category A) and emotional emptiness in “Everything grey” (category B). *Explanation of categories of Drawing acquisition: category A: Drawings were collected during inpatient stays at the University Medical Center Hamburg‐Eppendorf, Hamburg, Germany, in response to prompts designed to stimulate creativity, strengthen self‐confidence. Category B: Drawings were solicited via email through the patients supporting groups “German Parkinsons disease association” and “PingPongParkinson.” Category C: Drawings were collected in the Netherlands.

### 3.4 “Freezing and Falls”

The fourth overarching theme emerging from the artwork of individuals with PD is “Freezing and falls.” (Figure 4, Figure S2, lower row) This theme primarily captures the perceived vulnerability around falling during FoG, encompassing fear and anticipation of falling, subjective experiences of instability or loss of bodily control, as well as participants' accounts of and questions about actual falls and their consequences. Thus, the theme reflects the participants' lived experience of a perceived close relationship between freezing and falling, rather than establishing a biomechanical or causal relationship between FoG and individual falls. In these depictions, freezing is experienced not only as motor arrest, but also as a moment of precarious instability, standing at the threshold between movement and potential collapse. In these depictions, freezing is experienced not only as motor arrest, but as a moment of precarious instability, standing at the threshold between movement and collapse. The images convey vulnerability, fragmentation of bodily control, and the constant fear of falling. In his picture “Standing at the freezing edge,” R.L. portrays a figure positioned at the edge of a chasm. He explains: “The figure itself is somewhat layered, meant to be in motion with energy even though it stands still. This reflects what a person with Parkinson's experiences, the mind is moving and everything feels in motion, even if the body does not cooperate. The chasm is actually somewhat ambivalent, because the next step during a FoG doesn't work, as if the body is blocked or resisting, just like standing at the edge of a chasm.” The layered figure symbolizes internal dynamism despite external immobility. The chasm represents both the blocked next step and the looming risk of falling. Freezing thus becomes a liminal state, poised between intention and breakdown. O.T., in his artwork “The fear of breaking through freezing ice,” intensifies this imagery of instability: “The legs are not ‘one whole piece’ but broken into separate parts, hard to control, unsteady and the ground opens up in front of you, swallowing you when you fall through it during a freezing episode.” Here, the body itself appears fragmented. The legs are no longer experienced as a coherent unit but as disjointed parts, undermining balance and control. The image of breaking through ice and being swallowed by the ground powerfully conveys both physical collapse and existential fear. S.AM. reflects on the uncertainty surrounding freezing and falls: “Freezing: Sometimes consciously present … But are my numerous sudden falls also due to unconscious freezing?” Her question introduces an important dimension. Freezing may not always be perceived consciously, yet it may still precipitate falls. This highlights the clinical complexity of distinguishing freezing‐related falls from other causes of instability. It also emphasizes that FoG must always enter the differential diagnosis when individuals with PD report having sudden, unexplained falls. J.A. extends the perspective to a societal level in his cartoon “Come on folks this way.” He explains: “This image uses the concept that as the population ages, many frail people with Parkinson's who fall will be brought to a hospital.” His reflection situates individual freezing episodes within the broader context of aging, frailty, and healthcare systems. Across these artistic and narrative accounts, freezing is depicted as a moment of suspended motion at the brink of physical danger. Together, these descriptions portray FoG as a phenomenon that extends beyond immobility. It is experienced as a state of precarious balance, where blocked movement and gravitational pull converge. The fear of falling is not merely a consequence but an intrinsic component of the freezing experience itself, transforming each halted step into a potentially dangerous threshold.

**FIGURE 4:**
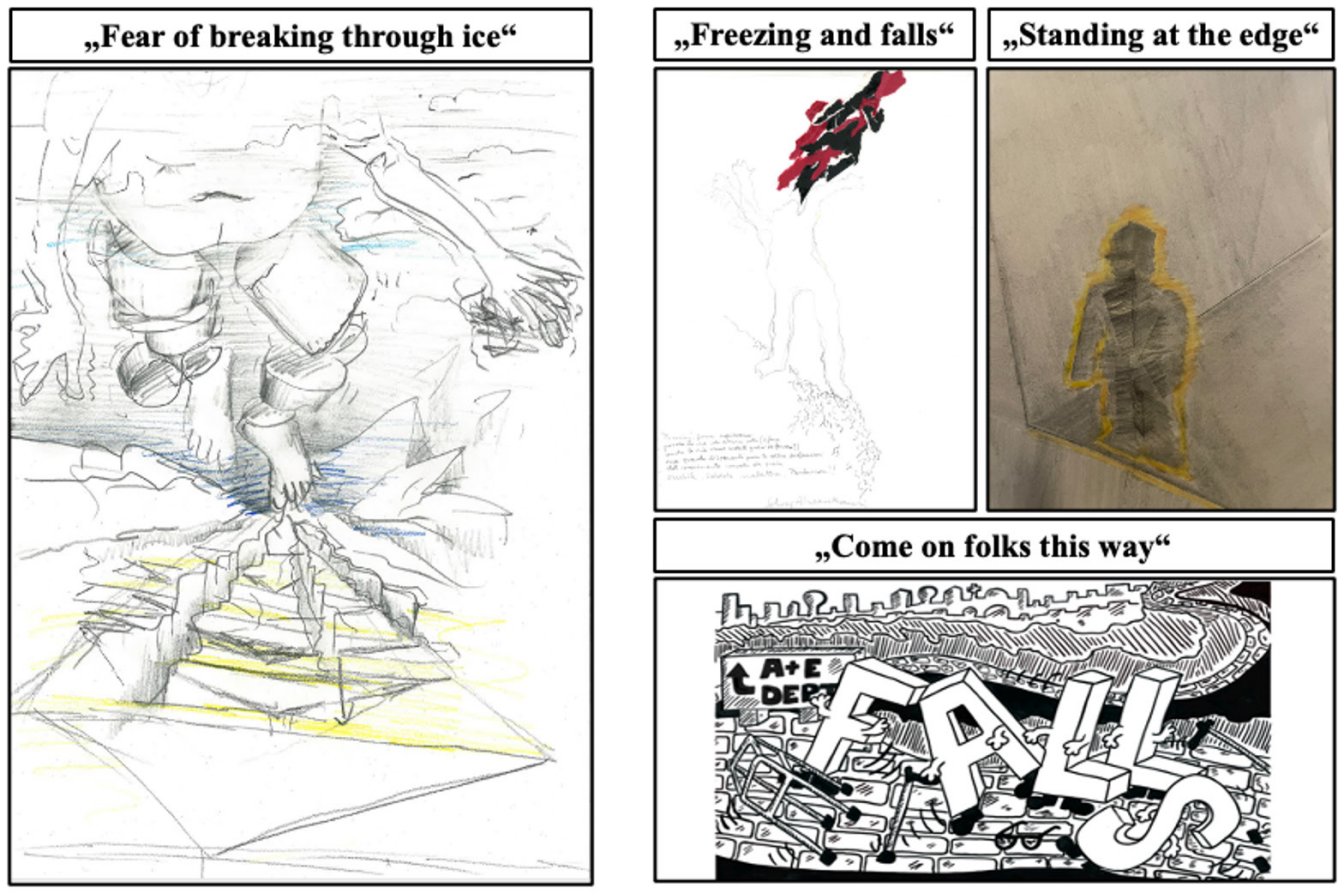
“Freezing and falls.” Visual expressions of the associated risk of falling during freezing of gait episodes in Parkinsons disease leading to fear in “Fear of breaking through ice” (category A), uncertainty in “Freezing and Falls” (category B), feelings of standing at a chasm “Standing at the edge” (category B), injury and potential hospitalization in “Come on folks this way” (category C). *Explanation of categories of Drawing acquisition: category A: Drawings were collected during inpatient stays at the University Medical Center Hamburg‐Eppendorf, Hamburg, Germany, in response to prompts designed to stimulate creativity and strengthen self‐confidence. Category B: Drawings were solicited via email through the patients supporting groups “German Parkinsons disease association” and “PingPongParkinson.” Category C: Drawings were collected in the Netherlands.

### 3.5 “Released From Freeze”

The fifth and final overarching theme inspired from the artworks can be summarized as “Released from freeze,” the therapeutic release and the joy of regained movement (Figure 5 and Figure S3). In contrast to the preceding themes of fixation, chaos, emotional explosion, and fear of falling, these images portray relief, vitality, and restoration. They emphasize the transformative moment when freezing resolves, whether spontaneously or through therapeutic intervention, and walking becomes fluid again.

**FIGURE 5:**
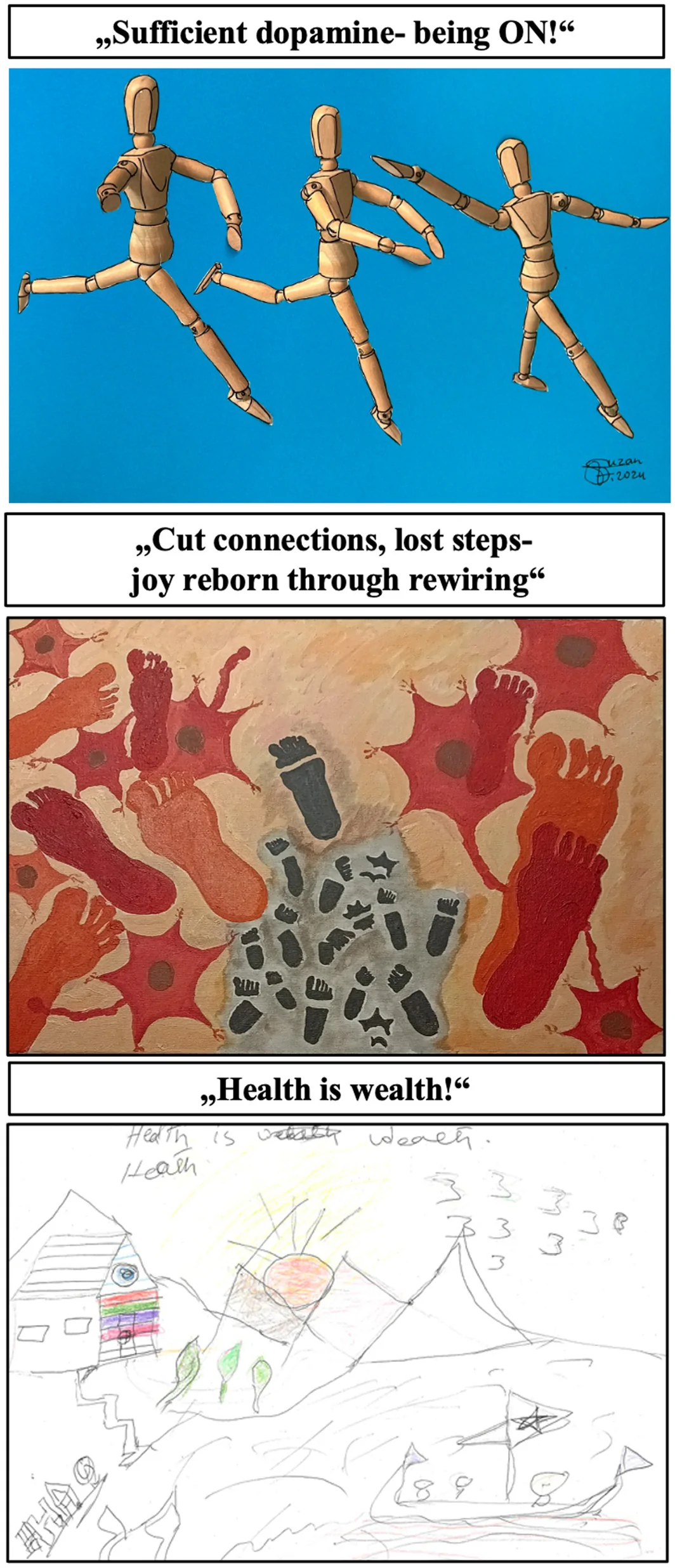
“Released from freeze.” Illustrative representations of the transition from freezing of gait to restored movement in Parkinsons disease. Patients portrayed the resolution of freezing as lightness in “Sufficient dopamine‐being On!” (category C), regained neural connection, vitality, and emotional rebirth in “Cut connection, lost steps‐joy reborn through rewiring” (category B), and transforming walking from immobilization and fear into freedom, celebration, and fluid motion in “Health is wealth!” (category A). *Explanation of categories of Drawing acquisition: category A: Drawings were collected during inpatient stays at the University Medical Center Hamburg‐Eppendorf, Hamburg, Germany, in response to prompts designed to stimulate creativity, strengthen self‐confidence. Category B: Drawings were solicited via email through the patients supporting groups “German Parkinsons disease association” and “PingPongParkinson.” Category C: Drawings were collected in the Netherlands.

S.F. describes this release as “Flying puppets,” jumping into the air when sufficient dopamine levels are present, the joyful “On” state. Her imagery conveys lightness, buoyancy, and regained agency. The body, previously rigid and constrained, becomes animated and liberated. The metaphor of puppets suggests that when dopamine is available, movement is once again possible, no longer blocked, but lively and expressive. B.S., in her artwork “Cut connections, lost steps – joy reborn through rewiring,” illustrates the neurobiological dimension of this transformation: “The picture I painted shows nerve cells and feet. The joyful colors, red and orange, symbolize movement, liveliness, and vitality. Then comes the freezing, which in my view only deserves the color black, which also stands for grief. My brain can no longer reach my feet, the connections between nerve cells and feet are severed. When the freezing is over, my life is like a celebration, how wonderful it is when I can walk.” Her depiction explicitly links freezing to disrupted neural connectivity and recovery to restored communication between brain and body. The shift from black to vibrant colors symbolizes the emotional and functional transition from immobility to movement. Walking, in this context, is not merely locomotion, it becomes celebration.

S.H., in his picture “Health is wealth!” frames walking with freezing as a journey marked by variability. “Walking with the risk of freezing is like a journey, sometimes like navigating a stormy sea, full of fear and unease, sometimes like a steep, exhausting mountain hike, and sometimes like strolling across a beautiful meadow to home when walking flows effortlessly.” He further painted “No FoG, full speed, highway to heaven!” (Figure S3), expressing the exhilaration of fluid gait when symptoms are controlled. Some individuals explicitly portrayed the relief of gait problems through levodopa therapy or deep brain stimulation, emphasizing the tangible impact of medical treatment in restoring FoG, gait, mobility, and quality of life (Figure S3). Across this final theme, freezing is contrasted with its resolution with the sudden lightness and joy of regained mobility, the restoration of brain–body communication and emotional celebration following motor release, a worthwhile therapeutic target.

## 4 Discussion

This study revealed five overarching themes that were presented in the visual artworks produced by individuals with PD on episodes of FoG: “Captured feet,” “Chaos,” “Emotional explosion,” “Freezing and falls,” and “Released from freeze.” Collectively, this work clarifies that FoG emerges not only as a motor phenomenon but as a multidimensional lived experience encompassing physical immobilization, cognitive overload, emotional escalation, existential threat, and, ultimately, profound relief when movement returns. The artistic representations reveal freezing as a deeply embodied and emotionally charged event, while also highlighting the hope and restoration made possible through therapeutic intervention.

By allowing individuals with PD to depict experiences and emotions associated with FoG, drawing can reveal aspects that may be difficult to articulate verbally and can complement structured questionnaires or clinical observation. Patient‐created art may offer a prompt for richer patient‐practitioner communication and may help clinicians explore the emotional and psychological dimensions of gait impairment in a person‐centered way. Whether incorporating artistic expression into clinical practice improves symptom evaluation, adherence, or treatment individualization requires a prospective study.

Several limitations should frame interpretation of these findings. The collection combined pre‐existing artworks, drawings sent after a call through patients' support groups and artworks elicited using open‐ended prompts, which may have shaped the metaphors and topics represented. Some individuals contributed multiple artworks, so the artworks are not statistically or analytically independent observations. The five themes arose from a multidisciplinary curatorial interpretive synthesis rather than a formal coded qualitative methodology, and no formal audit trail, coder‐reliability assessment, or member checking of the final thematic structure was undertaken. Although participants' own titles and explanations anchored interpretation, expert‐derived grouping may not fully reproduce each participant's intended meaning. Finally, linguistic and cultural context may influence metaphors and their translation; the procedures used for translation should therefore be considered when interpreting cross‐cultural similarities and differences.

The best closing sentence for this article lies in the drawing (Figure S3) and words of an individual with PD: “I have learned that we have ‘seven colors in life’ as the rainbow. When we miss one color from life, we are disabled. Freezing is the missing color for me. Freezing slows my steps, just as the German regional train ‘Deutsche Bahn’ experiences unexpected pauses. Movement is color, warmth, and vitality.”

“Love for all, FoG for none!”

We can unfortunately no longer ask him to confirm, but we are certain that Nir Giladi could not have agreed more.

## Author Contributions

**Monika Pötter‐Nerger:** investigation, conceptualization, methodology, writing – original draft, writing – review and editing. **Blanca T. M. Spee:** conceptualization, writing – review and editing, investigation. **Molly Zeitzschel:** investigation, conceptualization, writing – review and editing. **Neele Siems:** investigation, writing – review and editing, conceptualization. **Alessandro Gulberti:** writing – review and editing, investigation, conceptualization. **Erika Howe:** conceptualization, investigation, writing – review and editing. **Kaylena A. Ehgoetz Martens:** conceptualization, investigation, writing – review and editing. **Moran Gilat:** conceptualization, investigation, writing – review and editing. **Alice Nieuwboer:** conceptualization, investigation, writing – review and editing. **Bastiaan R. Bloem:** conceptualization, investigation, writing – review and editing.

## Ethics Statement

Ethical approval was obtained from the local ethical committee at Hamburg.

## Conflicts of Interest

The authors declare no conflicts of interest.

## Supporting information


**Figure S1:** Visual illustrations of perceived feet's captivation during freezing of gait with long time to unfreeze in “Time to defrost” (category C), “Feet bound by rubber bands” (category A), or the feeling of feet pulled down into the earth “Gravity of Gait” (category A). Patients drawings reveal feet's chaotic disobedience in “Insubordination” (category B), leading to perceived abandonement in “Left back….” (category B) and emotional storms in “Before, while, after FoG” or “If freezing was some kind of weather” (category A). *Explanation of categories of Drawing acquisition: category A: Drawings were collected during inpatient stays at the University Medical Center Hamburg‐Eppendorf, Hamburg, Germany, in response to prompts designed to stimulate creativity, strengthen self‐confidence. Category B: Drawings were solicited via email through the patients supporting groups “German Parkinson's disease association” and “PingPongParkinson.” Category C: Drawings were collected in the Netherlands.

**Figure S2:** Pictorial representations of “Stops” during freezing of gait with perception of FoG as “Barrier Walk” (category C) or “Red Barrier” (category A) particularly when sliding from the hypermobile state into the Off in “Hypermobile‐Off” (category C) eventually leading to falls in “Falling” (category C). *Explanation of categories of Drawing acquisition: category A: Drawings were collected during inpatient stays at the University Medical Center Hamburg‐Eppendorf, Hamburg, Germany, in response to prompts designed to stimulate creativity, strengthen self‐confidence. Category B: Drawings were solicited via email through the patients supporting groups “German Parkinson's disease association” and “PingPongParkinson.” Category C: Drawings were collected in the Netherlands.

**Figure S3:** Patient's created artworks on the relieve of freezing of gait by medication or deep brain stimulation. The regained joy after Fog resolution is perceived as “No FoG, Full Speed, Highway to heaven” (category A). Participants perceived the evolving, therapeutical relieve as a transition from FoG “Before Treatment”(category B), “During medication intake or programming” (category B) and “After effective treatment” (category B). The ultimate goal: “Love for all, FoG for none!” (category A). *Explanation of categories of Drawing acquisition: category A: Drawings were collected during inpatient stays at the University Medical Center Hamburg‐Eppendorf, Hamburg, Germany, in response to prompts designed to stimulate creativity, strengthen self‐confidence. Category B: Drawings were solicited via email through the patients supporting groups “German Parkinson's disease association” and “PingPongParkinson.” Category C: Drawings were collected in the Netherlands.

## Data Availability

The artworks and accompanying participant explanations constitute the primary qualitative data for this study. Requests for access to additional study materials are considered by the corresponding author.
